# Lipid-related residual risk and renal function for occurrence and prognosis among patients with first-event acute coronary syndrome and normal LDL cholesterol

**DOI:** 10.1186/1476-511X-10-215

**Published:** 2011-11-19

**Authors:** Kuo-Liong Chien, Hung-Ju Lin, Hsiu-Ching Hsu, Ming-Fong Chen

**Affiliations:** 1 Institute of Epidemiology & Preventive Medicine, National Taiwan University, Taipei, Taiwan; 2 Department of Internal Medicine, National Taiwan University Hospital, Taipei, Taiwan

**Keywords:** acute coronary syndrome, residual risk, dyslipidemia

## Abstract

**Background:**

We investigated relationship of low levels of high density lipoprotein cholesterol (HDL-C), high levels of triglycerides, and renal function for the odds, prognosis and survival following acute coronary events among patients with a first event and normal low density lipoprotein cholesterol levels.

**Results:**

A case-control study based on 557 patients and 1086 matched control subjects was conducted. Case patients were followed up for survival with a median of 1.9 years. Participants in the higher quintiles of HDL-C had lower odds to develop acute coronary events (the adjusted odds ratios were 0.24 for the second, 0.24 for the third, 0.10 for the fourth and 0.05 for the fifth quintile). Patients with normal glomerular filtration rate were at a lower risk for all-cause death. However, a reverse association between triglycerides and death risk was found: patients with higher triglycerides were at a lower risk for all-cause death (adjusted relative risk, 0.38 for triglycerides ranging from 82 to 132.9 mg/dL, and 0.14 for triglycerides > = 133 mg/dL).

**Conclusions:**

Low HDL-C was significantly associated with acute coronary events, and triglyceride levels as well as renal function were inversely related to all-cause deaths after the coronary event.

## Introduction

Lipid-related residual risk, including low levels of high density lipoprotein cholesterol (HDL-C) and high levels of triglycerides, has become a clinical target since the availability of aggressive low density lipoprotein cholesterol (LDL-C) lowering by statin treatment [1-4]. Evidence has shown about 70% increased relative risk among those with versus without low HDL-C and high triglycerides [5,6]. In addition, a substantial incremental risk for further cardiovascular events was still apparent even in patients with LDL-C levels less than 70 mg/dL who had high triglycerides and low HDL-C [7,8]. Residual risk profiles, including low HDL-C and high triglycerides among normal LDL-C, should be considered for prevention of coronary events [9,10]. It is therefore necessary to investigate the role of lipid-related residual risk, including low HDL-C and high triglycerides, for the risk of the occurrence of coronary events.

In addition, chronic kidney disease status, estimated by lower glomerular filtration rates, is highly prevalent in Taiwan, with a prevalence rate of 12% in Taiwanese adults [11]. Evidence has shown that an association between poor renal function and cardiovascular diseases is apparent [12,13]. In addition, the role of lipid residual risk has not been investigated concurrently with renal function status in previous studies. Moreover, the data on the residual risk profiles for all-cause death after coronary events are scanty.

Therefore, it appears important to evaluate the prevalence of lipid-related residual risk, determine its association with acute coronary syndrome, and investigate the prognostic factors for survival after the events in Taiwanese patients. We investigated associations between low HDL-C, high triglycerides, and renal function status, for odds for coronary events among patients who had suffered from first events and were within normal LDL-C levels in this retrospective case control study, matching for age, sex, and LDL-C levels. We also conducted a cohort follow-up to record their survival status.

## Results

### Basic distribution of case-control subjects

Table 1 presents the basic characteristics of the control subjects and acute coronary syndrome case patients. Compared with the matched control subjects, the case patients were more likely to be hypertensive, smokers, and more likely to have a history of diabetes, stroke, advanced chronic kidney disease and a higher age, systolic blood pressure, serum creatinine and estimated glomerular filtration rates. The distributions of sex, body mass index, triglycerides and LDL-C were not statistically different between the case subjects and controls.

**Table 1:** Basic characteristics of the study participants

	Control subjects, n = 1086		Case patients, n = 557		
	N	%	N	%	P
Gender					0.77
Men	800	73.7	414	74.3	
Women	286	26.3	143	25.7	
Hypertension	129	11.9	402	72.3	<.0001
Diabetes mellitus	51	4.7	213	38.3	<.0001
Smoking	102	9.4	163	29.3	<.0001
Alcohol drinking	600	55.3	144	25.9	<.0001
Old stroke	7	0.6	58	10.4	<.0001
Hypolipidemic medication	121	11.1	185	33.2	<.0001
Glomerular filtration rate, ml/min/1.73 m^2^					<.0001
< = 60	215	19.8	188	37.1	
60.1-90	787	72.5	246	48.5	
> 90	84	7.7	73	14.4	

	Mean	SD	Mean	SD	
Age, yr	64.3	10.6	67.5	9.6	<.0001
BMI, kg/m^2^	24.4	3.0	24.7	3.7	0.07
Systolic BP, mmHg	124.8	14.1	135.7	26.0	<.0001
Diastolic BP, mmHg	72.4	9.2	73.1	16.0	0.36
Total cholesterol, mg/dL	177.7	27.6	162.6	31.8	<.0001
Triglycerides, mg/dL	126.3	84.6	133.1	99.9	0.17
HDL cholesterol, mg/dL	44.5	11.2	34.7	11.9	<.0001
LDL cholesterol, mg/dL	97.5	22.1	95.5	23.0	0.10
Serum creatinine, mg/dL	1.12	0.45	1.52	1.47	<.0001
Estimated glomerular filtration rate, ml/min/1.73 m^2^	70.0	15.0	64.6	25.6	<.0001

BMI: body mass index; BP: blood pressure; HDL: high density lipoprotein; LDL, low density lipoprotein;

### Associations with lipid-related residual risk as well as glomerular filtration rate and the occurrence of acute coronary syndrome

The numbers in each quintile of triglycerides, HDL-C, and estimated glomerular filtration rates for the case subjects and the multivariable adjusted odds ratios and 95% confidence intervals are listed in Table 2. For HDL-C the higher the quintile, the lower the odds. Compared with those in the first quintile, participants in the higher quintiles of HDL-C had lower odds to have acute coronary events. The multivariable adjusted odds ratios (95% confidence interval [CI]) were 0.24 (0.15 to 0.37) for the second, 0.24 (0.15 to 0.38) for the third, 0.10 (0.05 to 0.18) for the fourth and 0.05 (0.03 to 0.11) for the fifth quintile (test for trend, *P *< 0.0001). Adjustment for clinical covariates did not appreciably change the association. For triglycerides, the age, sex-adjusted odds ratio for participants with the highest quintile was 1.55 (95% CI, 1.11 to 2.17), compared with those in the lowest quintile (test for trend, *P *= 0.031). However, the odds ratios for triglycerides attenuated modestly after multiple adjustments. With regards to glomerular filtration rate, participants in the higher quintiles were at lower odds for acute coronary syndrome events compared with the lowest quintile. However, the highest filtration rate quintile was significantly associated with the odds for acute coronary syndrome events after multiple adjustments (odds ratio, 1.92, 95% CI, 1.12 to 3.28), compared with those in the lowest quintile.

**Table 2:** The number of case control subjects according to lipid quintiles and adjusted odds ratios in the study participants

	Q1	Q2			Q3			Q4			Q5			P
HDL cholesterol, mg/dL														<.0001
Range	< 35	35-39.9			40-45.9			46-52.9			> = 53			
Median	30	37			42			48			60			
Control	191	222			235			211			227			
Cases	310	99			75			38			21			
Odds ratio		OR	95% CI		OR	95% CI		OR	95% CI		OR	95% CI		Trend
Model 1	1	0.28	0.21	0.38	0.19	0.13	0.26	0.10	0.07	0.15	0.05	0.03	0.09	<.0001
Model 2	1	0.30	0.22	0.43	0.23	0.16	0.33	0.12	0.08	0.19	0.06	0.04	0.11	<.0001
Model 3	1	0.24	0.15	0.37	0.24	0.15	0.38	0.10	0.05	0.18	0.05	0.03	0.11	<.0001
Triglycerides, mg/dL														0.38
Range	< 67	67-89.9			90-120.9			121-166.9			> = 167			
Median	53	78			104			140			220			
Control	211	221			216			216			222			
Cases	91	123			106			103			129			
Odds ratio		OR	95% CI		OR	95% CI		OR	95% CI		OR	95% CI		Trend
Model 1	1	1.33	0.95	1.86	1.20	0.85	1.69	1.21	0.86	1.71	1.55	1.11	2.17	0.031
Model 2	1	1.33	0.90	1.95	1.12	0.75	1.67	0.90	0.60	1.35	1.10	0.73	1.64	0.70
Model 3	1	1.12	0.67	1.86	0.87	0.52	1.45	0.56	0.32	0.98	0.76	0.45	1.30	0.12
Glomerular filtration rate														<.0001
Range	< 60	60-67.7			67.8-73.7			73.8-81.2			> = 81.3			
Median	48.0	64.8			71.7			77.3			89.1			
Control	217	217			218			217			217			
Cases	190	56			58			70			133			
Odds ratio		OR	95% CI		OR	95% CI		OR	95% CI		OR	95% CI		Trend
Model 1	1	0.32	0.22	0.46	0.32	0.22	0.46	0.42	0.30	0.59	0.84	0.61	1.16	0.06
Model 2	1	0.31	0.20	0.47	0.35	0.23	0.53	0.43	0.29	0.64	0.90	0.62	1.31	0.31
Model 3	1	0.39	0.21	0.73	0.49	0.27	0.88	1.01	0.58	1.77	1.92	1.12	3.28	0.010

HDL: high density lipoprotein; LDL, low density lipoprotein

Model 1: adjusted for age & gender

Model 2: additional body mass index, smoking and drinking status

Model 3: additional hypertension, diabetes and estimated filtration rates for lipids as well as triglycerides & HDL cholesterol for filtration rates;

To investigate obesity as an effect modifier for the association between triglycerides and acute coronary syndrome, we performed subgroup analysis according to body mass status (Table 3). Among participants with a lower body mass index (< 24.4 kg/m^2^), the odds of acute coronary syndrome increased as the triglyceride quintile increased (adjusted odds ratio, 1.77, 95% CI, 1.00 to 3.13 for the highest quintile, compared with those with the lowest triglyceride quintile); however, the direction was inverse for those with a higher body mass index (> = 24.4 kg/m^2^). The interaction between body mass index and triglycerides for the odds of coronary event was significant (*P *= 0.046).

**Table 3:** Subgroup analysis by obesity status* for the risk of acute coronary syndrome events in the study participants

	Q1	Q2			Q3			Q4			Q5			P	
Triglycerides														0.046	
Odds ratio		OR	95% CI		OR	95% CI		OR	95% CI		OR	95% CI		Test for trend	One quintile
BMI < 24.4	1	1.73	1.06	2.82	1.65	0.97	2.79	1.53	0.88	2.67	1.77	1.00	3.13	0.15	0.09
BMI > = 24.4	1	0.85	0.45	1.59	0.67	0.37	1.24	0.51	0.28	0.94	0.69	0.39	1.24	0.28	0.10

BMI: body mass index; OR: odds ratio; CI: confidence interval;

Interaction between triglycerides & BMI, *p *= 0.046

* BMI cutoff point = 24.4 kg/m^2^

### Predictive factors for mortality after acute coronary syndrome

**Figure 1:**
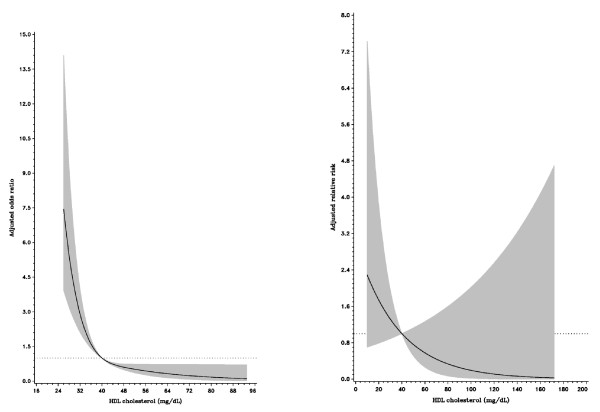
**Relationship between HDL-C concentrations and the risk of acute coronary syndrome event (left) and all-cause death after event (right)**. The multivariate adjusted relative risks were plotted as a function of the baseline HDL-C value with the 95% confidence bands shown as the shaded area.

**Figure 2:**
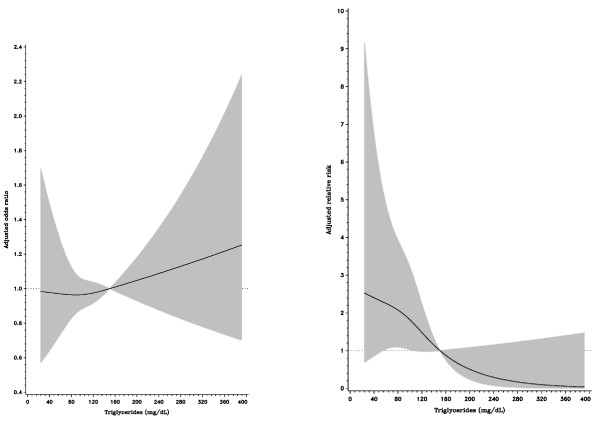
**Relationship between triglyceride concentrations and the risk of acute coronary syndrome event (left) and all-cause death after event (right)**. The multivariate adjusted relative risks were plotted as a function of the baseline triglyceride value with the 95% confidence bands shown as the shaded area.

**Figure 3:**
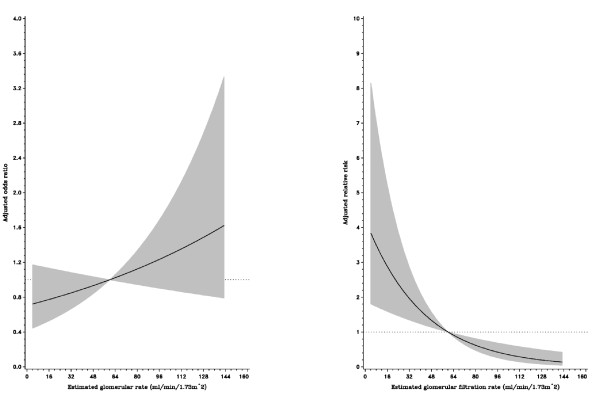
**Relationship between glomerular filtration rates and the risk of acute coronary syndrome event (left) and all-cause death after event (right)**. The multivariate adjusted relative risks were plotted as a function of the baseline glomerular filtration rate with the 95% confidence bands shown as the shaded area.

After an average of 2.1 years (median: 1.9, inter-quartile range: 1.1-3.0) of follow-up among 1086 patients, 39 cases died, including 10 due to cardiovascular deaths. Table 4 presents the results from a multiple Cox regression model for the risk of all-cause death after acute coronary syndrome events. We found that only glomerular filtration rates and triglycerides were significant predictors for mortality after acute coronary syndrome. Compared with those with a glomerular filtration rate < 60 ml/min/1.73 m^2^, patients with filtration rates ranging from 60 to 89.9 and > = 90 ml/min/1.73 m^2 ^were at a lower risk for all-cause death (adjusted relative risk, 0.19 [95% CI, 0.08 to 0.45] and 0.26 [95% CI, 0.06 to 0.19]). In addition, an reverse association between triglycerides and death risk for patients with first coronary events was found. Compared with those with low triglycerides (< 82 mg/dL), patients with high triglycerides had a lower risk for death (adjusted relative risk, 0.38, [95%CI, 0.18 to 0.83] for triglyceride levels ranging from 82 to 132.9 mg/dL, and 0.14 [95% CI, 0.05 to 0.43] for triglyceride levels > = 132 mg/dL). The spline associations between HDL-C, triglycerides and glomerular filtration rates were plotted accordingly. HDL-C was inversely associated with an acute coronary syndrome event, however, HDL-C was not related to death after the event (Figure 1). In contrast, high triglycerides were not related to the occurrence of a coronary event, although triglycerides were inversely related to death after the event (Figure 2). Finally, although glomerular filtration rate was not associated with coronary events, a high filtration rate was a protective factor for death after a coronary event (Figure 3).

**Table 4:** The multiple Cox regression model for the risk of all-cause death among patients with first acute coronary syndrome events and normal LDL cholesterol levels

Covariates	Relative risk	95% CI		P
Gender, women vs. men	0.77	0.35	1.73	0.53
Age, 60-74.9 vs. 50-59.9 yrs	1.70	0.47	6.23	0.42
Age, > = 75 vs. 50-59 yr	2.96	0.79	11.05	0.11
History of hypertension	1.16	0.50	2.68	0.73
History of type 2 diabetes	1.17	0.57	2.39	0.68
Glomerular filtration rate, 60-89.9 vs. 60, ml/min/1.73 m^2^	0.19	0.08	0.45	0.0002
Glomerular filtration rate, > = 90 vs. 60, ml/min/1.73 m^2^	0.26	0.06	1.19	0.08
HDL cholesterol, 38-47.9 vs. < 38 mg/dL	0.53	0.23	1.24	0.14
HDL cholesterol, > = 48 vs. < 38 mg/dL	0.18	0.03	1.37	0.10
Triglycerides, 82-132.9 vs. < 82 mg/dL	0.38	0.18	0.83	0.015
Triglycerides, > = 133 vs. < 82 mg/dL	0.14	0.05	0.43	0.001

HDL: high density lipoprotein; LDL, low density lipoprotein

## Discussion

In this matched case-control and cohort study among patients with first-event acute coronary syndrome and normal LDL-C levels, we demonstrated that increasing HDL-C was associated with lower odds for acute coronary syndrome events. HDL-C has been proven to be a protective factor for coronary artery disease. Epidemiological studies such as the Framingham Study [14] and the Prospective Cardiovascular Münster (PROCAM) study [15] have established that a low HDL-C is an independent risk factor for coronary events. Among the patients hospitalized due to coronary artery disease, low HDL-C was highly prevalent, with a prevalence rate of up to 55%[16]. In addition, a low HDL-C level implies a high ratio of atherogenic apoB-containing lipoproteins to atheroprotective apoA-I [17,18]. Low HDL-C is prevalent across all LDL-C levels, particularly so among patients with LDL-C levels less than 70 mg/dL, even with statin therapy (64%)[18]. Our study implies a significant inverse association of HDL-C for the odds of acute coronary syndrome among patients with normal LDL-C levels.

However, our data did not support an independent association between triglycerides and coronary events, after controlling for obesity and clinical disease status. A meta-analysis based on 10, 158 cases of coronary events from 262, 525 subjects in 29 studies showed a summarized odds ratio of 1.72 (95%CI 1.56-1.90) in a comparison of individuals in the top third with those in the bottom third oftriglycerides [19]. However, the role of fasting triglycerides as the risk factor was still controversial. In addition, evidence has favored postprandial triglycerides, instead of fasting triglycerides, as the better predictor for coronary events [20,21]. Although past evidence on combined low HDL-C and high triglycerides, "atherogenic dyslipidemia" predicting cardiovascular risk are available, our results did not support this hypothesis regarding the joint associations between HDL-C, triglycerides, and acute coronary events. The association was greatly attenuated after obesity and renal function were incorporated into the model, implying that the association between dyslipidemia and acute coronary events were modified according to the obesity and renal function status.

Evidence showing robust associations between increasing fasting trigylcerides and declining HDL-C/tirglyceride ratios with cardiovascular risk has been derived from prospective studies based on communities and hospitals [9,19,22,23], and our hosptial-based case-control study results were consistent to previous findings. In addition, our further follow-up study showed that unexpected findings for the risk of all-cause deaths among patients who suffered from acute coronary events and had a normal LDL-C level. The patterns of statistical significance of the associations between triglycerides, odds for acute coronary syndrome, and mortality risk may be attributed from the following reasons. First, lipid-related residual risk is associated with acute coronary syndrome events, after which the patients develop wasting and then low triglycerides may affect survival [24]. Recent evidence has shown that traditional atherosclerotic risk factors, such as obesity and lipid levels, are inversely associated with survival in patients with congestive heart failure and chronic diseases [24-26]. Second, body fat changes after the event, such as obesity distribution, inflammatory processes and endotoxin-lipoprotein interaction, may contribute to the role of triglycerides, which are highly associated with obesity status, as an inverse relationship to death among patients with chronic diseases [27]. The interrelationshipe between obesity and triglycerides after coronary events may account for the attenuation of the association between triglyceride levels and coronary risk.

Our previous study based on healthy adults showed that renal function deterioration was related to cardiovascular and all-cause death mortality, and that the effect was additive with the metabolic syndrome components [13]. In addition, a poor prognosis, including increased cardiovascular disease mortality, is associated with renal failure in chronic diseases [28-31]. The present study further demonstrated that poor renal function is also a poor prognostic factor for patients after acute coronary syndrome events. Therefore, identifying poor renal function and aggressively controlling the progress of renal function deterioration is crucial for improving the survival in patients experiencing a first coronary event.

This study has some limitations. First, our results are only applicable to middle-age and older patients with a normal LDL-C level due to the study design. Second, the problem of un-comparability of statin usages between cases and controls (20% in case patients and 8% in control subjects) may bias the association between lipid profiles and coronary events. Third, the follow-up period was relatively short, so the results are suitable for short-term prognosis and survival. In addition, we didn't specify the cause-specific death in the outcome assessment because of limited death cases due to cardiovascular events. Assessing all-cause mortality after acute cardiovascular events is a feasible way to evaluate the prognosis for secondary prevention [32].

## Conclusion

In conclusion, low HDL-C was significantly associated with acute coronary events, and triglyceride levels as well as renal function were inversely related to all-cause deaths after the coronary events. Further secondary prevention studies on the relationship between lipids, renal function and coronary events may be warranted.

## Methods

### Study design and study participants

We divided this study into two parts. The first was a retrospective matched case-control study designed to examine the association between low HDL-C and high triglycerides, and the occurrence of acute coronary syndrome. The second was a longitudinal cohort study, following up the patients with acute coronary syndrome until December 31, 2009. The protocol was approved by the IRB, National Taiwan University Hospital, and written informed consent was not obtained due to chart review and un-labelling participants' identificiation.

### Matched case-control study

The matched case-control study design was modified from the protocol of a multinational, multicenter case-control study [33]. In brief, patients hospitalized due to acute coronary syndrome, including unstable angina, ST elevated myocardial infarction and non-ST elevated myocardial infarction, from 2006 to 2009 were included. From a retrospective chart review, patients with high LDL-C (> = 130 mg/dL) were excluded and we limited the age range to 50 to 85 years. Cases were defined as patients with a first coronary event (acute coronary syndrome, including myocardial infarction) admitted to intensive care units or explored in catheterization labs. A total of 557 case patients were recruited. Diagnosis was regarded as established if supported by electrocardiography criteria and appropriate cardiac biomarkers, otherwise diagnosis was considered as not definitively confirmed. The controls selected for the case-control study were participants free of coronary events being hospitalized for health checkups in the same hospital during the same period. The 1:2 matched controls (n = 1086) were randomly selected from the participants, with matching factors for age (50-60, 60-75, and > = 75 yrs), sex, and LDL-C level (< 70, 70-100, 100-130 mg/dL).

### Prospective cohort study

The second part of this study was a prospective cohort study on the case patients. Information on mortality and causes of death were obtained by linking the identification numbers of the study subjects to a national databank provided by the National Health Administration, which was updated to the end of 2009. We defined all-cause death according to the death codes from the 9^th ^or 10^th ^versions of the International Classification of Diseases (ICD).

### Clinical and laboratory measurements

Data were collected family history, lifestyle habits, and medical history, as well as anthropometric measurements, such as body weight, and body height, when available. Clinical variables were collected for each subject consisting of measures of body mass index, lipid profiles, blood pressures and glucose levels. Baseline hypertension and diabetes mellitus status were checked from the chart review.

We defined the baseline lipid profiles during the examination as those within the first 8 hours following onset of acute coronary syndrome. Lipid levels were measured in the control participants when they received the health checkup during the fasting status. Procedures for blood sampling and analytic methods were the same in case patients and control subjects and were performed as previously described [34]. In brief, serum total cholesterol levels were measured using the CHOD-PAP method (Boehringer Mannheim, Germany) while HDL-C was measured following precipitation of apolipoprotein B-containing lipoproteins, with phosphotungstic acid and magnesium ions (Boehringer Mannheim, Germany)[35]. LDL-C levels were calculated if triglyceride levels were below 400 mg/dL. Triglycerides concentrations were measured by the GPO-DAOS method (Wako Co., Japan). All of the biochemical measurements, including the aforementioned lipid, uric acid, and creatinine concentrations, were measured using a Hitachi 7450 automated analyzer (Hitachi, Japan). All of the sample measurements were carried out in a single hospital. The coefficients of variation for the above measurements were around 5%.

Estimated glomerular filtration rates were calculated from serum creatinine measurements with the abbreviated MDRD equation [36]: $\text{eGFR }\left(\text{ml}∕\text{min}∕\text{1}.\text{73}{\text{m}}^{\text{2}}\right)=\text{186}\times \left[\left(\text{serum creatinine in mg}∕\text{dl}\right)**\text{1}.\text{154}\right]\times \left[\left(\text{age in years}\right)**0.\text{2}0\text{3}\right]\times \left(0.\text{742 if female}\right)$

### Statistical analysis

All data were presented as mean and standard deviation for continuous variables and contingency tables for categorical data, and were listed by status of case patient and control subject. The t-test was used to test the differences between case patients and control subjects. Participants were categorized on the basis of quintiles of HDL-C, triglycerides, LDL-C and estimated glomerular filtration rate from the control subjects.

Multiple logistic regression models to adjust for potentially confounding variables, including age, sex, estimated glomerular filtration rate, body mass index (continuous variable), alcohol intake (nondrinker/current), smoking (yes/no), baseline hypertension (yes/no) and type 2 diabetes mellitus (yes/no) were applied to estimate the odds ratios and 95% confidence intervals (CI) by quintiles of HDL-C and triglycerides. In addition, to test for linear trends across lipid marker categories, we used the median lipid profile levels within quintiles as a continuous variable. Moreover, we examined whether the association between triglycerides and acute coronary syndrome odds differed according to weight status. Likelihood ratio tests were used to compare the model with the interaction terms and the model without the interaction terms. We also tested the goodness-of-fit for the model by using the Hosmer and Lemeshow test, [37] and the goodness-of-fit test was acceptable. Finally, we conducted joint analyses to evaluate potential additive associations of baseline HDL-C and triglycerides using the tertiles in the control subjects as the cutoff.

With regards to the cohort follow-up study, we constructed a multivariable Cox proportional hazards model to incorporate possible clinical variables in one model. To test the proportionality assumption in the Cox regression model, we generated the time dependent covariates by creating interactions of the covariates and a function of survival time and included them in the Cox model [38]. The proportionality assumption for each covariate and overall model in the multiple Cox model were not rejected (all *P *values > 0.3).

In addition, we examined the non-linear relationship between lipids as well as filtration rates and the risk of coronary events and the survival non-parametrically with restricted cubic splines [39]. Tests for non-linearity used the likelihood ratio test, comparing the model with only the linear term to the model with the linear and the cubic spline terms. The relationships between lipids and outcomes were plotted accordingly.

All statistical tests were two-tailed with a type I error of 0.05, and *P *values < 0.05 were considered statistically significant. Analyses were performed with SAS software version 9.1 (SAS Institute, Cary, NC).

## Conflict of interest statement

The authors declare that they have no competing interests.

## Authors' contributions

KLC participated in collecting the data, writing the draft and performing statistical analysis. HJL carried out in collecting the data and revised the draft. HCH participated in performing the laboratory data and quality controlling. MFC participated in revising the draft and supervising the study. All authors read and approved the final manuscript.
